# Comparative Cost-Effectiveness Analysis of Respiratory Syncytial Virus Vaccines for Older Adults in Hong Kong

**DOI:** 10.3390/vaccines11101605

**Published:** 2023-10-17

**Authors:** Yingcheng Wang, Ginenus Fekadu, Joyce H. S. You

**Affiliations:** School of Pharmacy, Faculty of Medicine, The Chinese University of Hong Kong, Hong Kong SAR, China; yasminewyc@link.cuhk.edu.hk (Y.W.); mekonenginenus@link.cuhk.edu.hk (G.F.)

**Keywords:** respiratory syncytial virus, respiratory infection, vaccination, older adults, cost-effectiveness analysis

## Abstract

Two respiratory syncytial virus (RSV) vaccines (AREXVY^®^ and ABRYSVO^®^) were recently approved for older adults in the US. This study aimed to evaluate the cost-effectiveness of AREXVY^®^ and ABRYSVO^®^ from the Hong Kong public healthcare provider’s perspective. A two-year decision-analytical model was developed to examine the outcomes of a single RSV vaccination (AREXVY^®^ or ABRYSVO^®^) compared to no vaccination. Primary outcomes included RSV-related health outcomes, direct medical costs, quality-adjusted life-year (QALY) loss, and incremental cost per QALY (ICER). RSV vaccines are not yet marketed in Hong Kong, base-case analysis, therefore, benchmarked US RSV vaccine prices at 4 levels (25%, 50%, 75%, 100%). AREXVY^®^ and ABRYSVO^®^ (versus no vaccination) gained 0.000568 QALY and 0.000647 QALY, respectively. ICERs of ABRYSVO^®^ (26,209 USD/QALY) and AREXVY^®^ (47,485 USD/QALY) were lower than the willingness-to-pay threshold (49,594 USD/QALY) at 25% US vaccine price. The RSV attack rate was a common influential factor at all vaccine price levels. The probabilities of AREXVY^®^ and ABRYSVO^®^ to be most cost-effective were 0.10% and 97.68%, respectively, at 25% US vaccine price. Single vaccination of ABRYSVO^®^ or AREXVY^®^ for older adults appears to gain QALYs over 2 years in Hong Kong. The cost-effectiveness of AREXVY^®^ and ABRYSVO^®^ is subject to vaccine price and RSV attack rate.

## 1. Introduction

Respiratory syncytial virus (RSV) is a common cause of respiratory tract illnesses [1]. In 2015, it was estimated that there were 1.5 million RSV-associated acute respiratory infection (ARI) episodes in older adults in industrialized countries, with 14.5% requiring hospitalization [2]. In Hong Kong, the hospitalization incidence and mortality attributed to common respiratory viruses indicated that RSV was associated with a higher mortality rate than influenza [3,4], and RSV was a common cause of death among hospitalized elderly [5,6]. Since the relaxation of COVID-19 precautions, the incidence of respiratory infections has increased with SARS-CoV-2, influenza, and RSV circulating together. This phenomenon is referred to as “triple-demic” when infections caused by these three types of viruses surge at the same time period [7,8]. The symptoms for infections caused by each virus are similar, and vaccines against SARS-CoV-2 infection and influenza infection have been recommended to different population groups for several years, but only recently have RSV vaccines been authorized in certain regions.

Two RSV vaccines were recently approved by the US Food and Drug Administration (FDA) in May 2023 for individuals aged 60 years and older: AREXVY^®^ (GlaxoSmithKline Biologicals, Durham, NC, USA) (an adjuvanted RSV prefusion F protein vaccine) and ABRYSVO^®^ (Pfizer Inc., New York, NY, USA) (a bivalent RSV prefusion F vaccine) [9]. The US Advisory Committee on Immunization Practices (ACIP) recommended that older adults aged ≥60 years receive a single dose of RSV vaccine [10]. AREXVY^®^ is a combination of an RSVPreF3 antigen (120 μg) and AS01_E_ adjuvant system, designed to induce a humoral and cellular immune response in older adults [11]. ABRYSVO^®^ is a non-adjuvanted formulation with 60 μg of RSV A strain and 60 μg of RSV B strain [12,13]. Both vaccines demonstrated substantial efficacy in risk reduction of RSV-associated lower respiratory tract illness (LRTD) by 66.7% (96.66% CI: 28.8–85.8%) to 82.6% (96.95% CI: 57.9–94.1%) and RSV-associated ARI by 62.1% (95% CI: 37.1–77.9%) to 71.7% (95% CI: 56.2–82.3%) in phase three clinical trials (follow-up period 6–7 months) in older adults [13,14].

Despite the positive clinical findings of RSV vaccines for older adults, the cost-effectiveness of implementing an RSV vaccination program in Hong Kong has yet to be examined. To provide information to decision-makers on policy planning and resource allocation for the RSV vaccination program, this study aimed to evaluate the comparative cost-effectiveness of the two RSV vaccines, AREXVY^®^ and ABRYSVO^®^, for adults aged 60 years and older from the perspective of public healthcare providers in Hong Kong.

## 2. Method

### 2.1. Model Design

**Figure 1:**
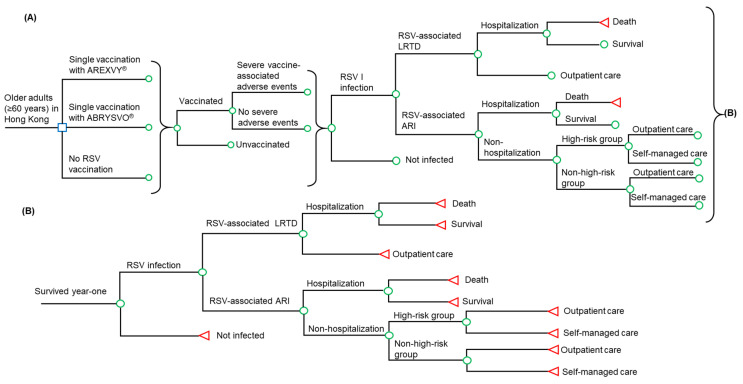
Simplified decision-analytical model for RSV vaccination in Hong Kong older adults (age ≥60 years). (**A**) year one post-vaccination, (**B**) year two post-vaccination. RSV: respiratory syncytial virus; LRTD: lower respiratory tract illness; ARI: acute respiratory infection. Square symbol: decision node, three branches on the right side of the decision node represent three strategies examined by the model; Circular symbol: chance node, represents the events characterized by event-specific probabilities; Triangle symbol: terminal node, represents the end of the model pathway.

A decision tree model (Figure 1) was developed to simulate the clinical and health-economic outcomes of the two RSV vaccines in a hypothetical cohort of adults aged 60 years and older in Hong Kong. Three RSV vaccination strategies were compared in the model: (1) vaccinated with a single dose of AREXVY^®^, (2) vaccinated with a single dose of ABRYSVO^®^, and (3) no RSV vaccination. A decision tree is a form of decision-analytical model in which individuals proceed through different health events or states over a fixed time horizon. The time horizon was two years in the present model, and the model timeframe aligned with the follow-up period of RSV vaccine efficacy studies in year one post-vaccination [13,14] and year two post-vaccination [15,16]. Primary model outcomes included RSV infection, RSV-associated hospitalization, death, direct medical cost, and quality-adjusted life-year (QALY) loss. QALYs are the most commonly used composite measure of health outcomes in economic evaluation, which combines the quality of life and survival. The methods of calculating QALY loss and costs for each vaccination option are described in Section 2.3 “utility inputs” and Section 2.4 “cost inputs”, respectively.

In the two vaccinated groups, the individuals were offered the RSV vaccine, and those who were vaccinated might experience vaccination-related severe adverse events. In the no RSV vaccination group, the individuals were all unvaccinated. All individuals, vaccinated or not, are susceptible to RSV infection during the first year post-vaccination. The infected patients might experience RSV-associated LRTD or ARI. The treatment venue of respiratory tract infections is driven by the severity of the infection, and patients with RSV-associated LRTD receive outpatient or inpatient care. Patients with RSV-associated ARI might be hospitalized, or treated outside the hospital (outpatient care or self-managed care). A healthcare-seeking behavior study showed that individuals with high-risk chronic conditions (versus non-high-risk ones) are more inclined to seek medical care for mild illnesses such as ARI [17]. The high-risk group was defined as individuals with chronic diseases, such as diabetes, cardiovascular diseases, or chronic pulmonary disease. The ARI patients (who were treated outside the hospital) were, therefore, further differentiated as high-risk or non-high-risk patients and the corresponding care-seeking behaviors (for outpatient care or self-managed care). All hospitalized RSV patients (with LRTD or ARI) might survive or die. The individuals who survived year one post-vaccination are susceptible to RSV infection in year two post-vaccination. Similar to year one, the individuals who were infected during year two might experience RSV-associated LRTD or ARI and might be hospitalized or treated outside the hospital (outpatient care or self-managed care).

### 2.2. Clinical Inputs

A MEDLINE search was performed over the period of 2000–2023 for the parameter value of model inputs. The search keywords mainly included “RSV infection”, “RSV vaccine”, “RSV disease burden”, “older adults”, “RSV hospitalization”, and “RSV mortality”. The detailed search strategies and the flowchart of the selection process are illustrated in the Supplementary Materials Table S1 and Figure S1, respectively. The selection criteria of clinical studies were: (1) reports written in English; (2) older adults (≥60 years) with RSV infection; and (3) treatment outcomes of RSV infection were reported. Studies containing data relevant to model inputs were included. When multiple sources were relevant for model input, a weighted average (mean value) was used as the base-case value [18]. All model inputs are listed in Table 1.

RSV is not in the public health surveillance program of Hong Kong, and the annual attack rate of RSV was, therefore, approximated from the findings of a meta-analysis in adults aged ≥60 years in high-income countries (Belgium, Canada, Czech Republic, Estonia, France, Germany, Japan, Mexico, Norway, Poland, Romania, Russia, the Netherlands, Taiwan, UK, and US). The pooled attack rate of 14 studies was reported to be 1.62% (95% CI: 0.84–3.08%; range: 0.07–7.21%) [19]. The sensitivities of RSV tests are less than 100% and underestimate the burden of RSV cases. The reported RSV attack rate in the meta-analysis was, therefore, adjusted by a multiplier (2.19, 95% CI: 1.72–2.97) to correct for under-detection of RSV infections associated with polymerase chain reactions [20]. The proportion of ARI (84.2%) among RSV infections was estimated from the prevalence of upper respiratory tract infections among RSV infections reported by an epidemiology study of RSV infections in Mainland China [21]. The RSV-associated hospitalization and mortality rates in Hong Kong were reported by a 15-year retrospective study, and the findings of the older adults were reported for 3 age groups (60–64 years, 65–74 years, and ≥75 years) [5]. To approximate the hospitalization rate among RSV-infected older adults, the number of infected and hospitalized patients was estimated using Hong Kong age-specific populations [22], adjusted RSV attack rate, and adjusted age-specific hospitalization rate among the population. The distribution of the hypothetical cohort (≥60 years) in the three age groups (60–64 years, 65–74 years, and ≥75 years) was retrieved from the Hong Kong population data [22]. Similarly, the mortality rate among hospitalized individuals in each age group was approximated from the estimated numbers of hospitalization and mortality cases using the age-specific population [22], adjusted age-specific hospitalization rate, and mortality rate [5].

The proportions of patients who sought medical care among high-risk (53.57%) and non-high-risk (17.39%) older adults were adopted from the findings of a prospective RSV epidemiology surveillance study in the US [23]. The proportions of high-risk individuals among older adults (47%) adopted the value of a model input (derived from the findings of an RSV disease burden study) previously applied in a health economic evaluation of a hypothetical RSV vaccine for older adults in the UK [24,25]. 

The model adopted the published vaccination coverage data for seasonal influenza (48.2%) of Hong Kong in 2022–2023 as a proxy for potential RSV vaccination coverage [26]. Vaccine efficacies (VE) against RSV-associated LRTD and ARI were retrieved from findings of phase 3 clinical trial data and data reported at the 2023 ACIP meeting [15,16]. For AREXVY^®^, the VE against RSV in season one decreased from 87.5% (95% CI: 58.9–97.6%) to 52.9% (95% CI: 0–81.2%) against LRTD, and from 79.0% (95% CI: 54.3–91.5%) to 27.8% (95% CI: 0–60.4%) against ARI in season two [14,27]. The VE of ABRYSVO^®^ against RSV in season one also decreased from 84.6% (95% CI: 32.0–98.3%) to 75.0% (95% CI: −25.3–97.4%) against LRTD, and from 65.2% (95% CI: 36.0–82.0%) to 55.0% (95% CI: −3.4–82.0%) against ARI in season two [13,27]. The RSV-associated event rate in vaccinated older adults was estimated using the following formula: unvaccinated RSV-associated event rate × (1 − VE against RSV-associated event). Severe adverse events after vaccination were considered in the model, which were defined as Grade 3 solicited local reactions (injection site pain, redness, and swelling) or systemic reactions (such as fatigue, fever, headache, gastrointestinal symptoms, and muscle pain). The incidence rates of severe adverse events in AREXVY^®^ and ABRYSVO^®^ were 3.8% and 1.0%, respectively [10].

### 2.3. Utility Inputs

The expected RSV-associated QALY loss was calculated using the utility decrements of RSV-related health events and the patient time spent in each event. The utility decrement was estimated by the difference in the age-specific utility score of uninfected individuals and the utility of RSV-related health events. The events included in the model were: (1) vaccine-related severe adverse events; (2) self-managed care; (3) outpatient care; and (4) hospitalization. The QALY loss of severe adverse events was 0.000677, reported by a single-arm clinical trial of Grade 3 reactogenicity after the first dose of recombinant vaccine [28]. An observational cohort study estimated the health-related quality of life among European community-dwelling older adults (aged ≥60 years) with RSV infection treated as outpatients. The collected data covered symptom onset to a symptom-free state by EuroQol 5-Dimensions 5-Levels (EQ-5D-5L). The reported utility values of uninfected (0.896), self-managed care (0.82), and outpatient care (0.75) were adopted in the model [29]. A prospective cohort study (in 12 countries: Australia, Argentina, Brazil, Canada, France, Germany, Japan, Malaysia, Mexico, Republic of Korea, South Africa, and USA) of health-related quality of life on adult patients hospitalized with acute respiratory tract infections, including RSV cases, was used. The utility score collected by EQ-5D-5L (0.576) was adopted as a utility for hospitalization in the present model [30]. The duration of illness was applied to approximate the time spent in self-managed care and outpatient care. The mean length of illness for RSV outpatient/self-managed care (15.5 days) was reported by a prospective RSV epidemiology surveillance study in the US [23]. The length of hospital stay (12 days) for RSV inpatient care was retrieved from the findings of a retrospective cohort study on RSV-hospitalized adults in Hong Kong [3]. The QALY loss due to RSV-associated death was estimated using the age of hypothetical individuals, age-specific life expectancy data from the Hong Kong life table in 2022, and age-specific health utilities [31,32]. In the model, the mid-point value of the age range was applied as the base-case age for the age group (62 years for 60–64 years, 70 years for 65–74 years, and 80 years for ≥75 years). The RSV-associated QALY loss was discounted to the current year with an annual discount rate of 3%. 

### 2.4. Costs Inputs

The cost analysis considered the direct medical costs, including RSV vaccines, treatment (self-managed care, outpatient care, and hospitalization) for RSV-associated LRTD/ARI, and vaccine-related severe adverse events. The cost per general outpatient clinic visit and the cost per hospital day (at general medical wards) were estimated using the 2023 charges listed by the Hospital Authority (the public healthcare provider in Hong Kong) [33]. The base-case number of clinic visits for RSV outpatient care was conservatively assumed to be at least one time and further examined over a range of 1–3 visits in the sensitivity analysis. RSV-infected patients who did not seek care were self-managed with over-the-counter products (estimated to be USD 13; USD 1 = HKD 7.8). The management of vaccine-associated severe adverse events was assumed to be at least one time of outpatient clinic visit. The economic evaluations of RSV vaccines in the US reported at the 2023 ACIP meeting had applied USD 270 and USD 200 as the base-case cost per vaccination for AREXVY^®^ and ABRYSVO^®^, respectively [27]. The RSV vaccines are yet to be marketed in Hong Kong and the pricing information is, therefore, unavailable. The base-case analysis in the present model benchmarked the US prices for AREXVY^®^ and ABRYSVO^®^ at four different levels is used in order to examine the outcomes and influential factors on cost-effectiveness at various vaccine price levels. The four price levels for AREXVY^®^ and ABRYSVO^®^ were 25% (USD 67.5 and USD 50), 50% (USD 135 and USD 100), 75% (USD 203 and USD 150), and 100% (USD 270 and USD 200) of the vaccine price used in the US economic models. All costs were discounted to the current year with an annual discount rate of 3%.

**Table 1:** Model inputs.

Parameters	Base-Case Value	Range for Sensitivity Analysis	Distribution	Reference
**Clinical inputs**				
Proportion of Hong Kong adults aged ≥60 years in				[22]
60–64 years (Base-case: 62 years)	28.64%	28.28–30.19%	Dirichlet	
65–74 years (Base-case: 70 years)	42.80%	41.13–43.33%	Dirichlet	
≥75 years (Base-case: 80 years)	28.56%	28.29–28.68%	Dirichlet	
RSV attack rate	1.62%	0.7–7.21%	Beta	[19]
Proportion of RSV–ARI among RSV infections	84.2%	67.4–95.0%	Beta	[21]
RSV hospitalization rate (per 10,000 population)				[5]
60–64 years	1.054	0.836–1.254	Triangular	
65–74 years	2.090	1.672–2.508	Triangular	
≥75 years	10.095	8.070–12.114	Triangular	
RSV mortality rate (per 10,000 population)				[5]
60–64 years	0.0645	0.0516–0.0773	Triangular	
65–74 years	0.1423	0.1138–0.1708	Triangular	
≥75 years	0.8263	0.6610–0.9916	Triangular	
Multiplier for under-detection of RSV by:				[20]
Polymerase chain reaction	2.19	1.72–2.97	Normal	
Rapid antigen assays	3.47	2.59–4.99	Normal	
Proportion of high-risk group among older adults	0.47	0.376–0.564	Beta	[24,25]
Probability of seeking medical care				[23]
High-risk group	53.57%	42.86–64.29%	Beta	
Non-high-risk group	17.39%	13.91–20.87%	Beta	
Vaccine coverage	48.2%	38.56–57.84%	Triangular	[26]
Vaccine efficacy				
AREXVY^®^				[14,34]
Season 1				
RSV-ARI	79%	54.3–91.5%	Beta	
RSV-LRTD	87.5%	58.9–97.6%	Beta	
Season 2				
RSV-ARI	27.8%	0–60.4%	Beta	
RSV-LRTD	52.9%	0–81.2%	Beta	
ABRYSVO^®^				
Season 1				[13,34]
RSV-ARI	65.2%	36–82%	Beta	
RSV-LRTD	84.6%	32–98.3%	Beta	
Season 2				
RSV-ARI	55%	0–82%	Beta	
RSV-LRTD	75%	0–97.4%	Beta	
Probability of severe adverse events				
AREXVY^®^	3.8%	2.9–5.6%	Beta	[10,14]
ABRYSVO^®^	1.0%	0.7–1.2%	Beta	[10,13]
**Cost inputs (USD)**				
Vaccine price per vaccination in the US				[27]
AREXVY^®^	270	/	/	
ABRYSVO^®^	200	/	/	
Cost of inpatient care (per day)	654	523–785	Gamma	[33]
Cost of outpatient care (per clinic visit)	57	46–68	Gamma	[33]
Cost of self-managed care (per episode)	13	10–15	Gamma	Assumption
Length of illness for RSV outpatient/self-managed care (days)	15.5	11.6–18.4	Normal	[23]
Length of hospitalization for RSV inpatient care (days)	12	5–14	Normal	[3]
Number of clinic visits for RSV outpatient care	1	1–3	Triangular	Assumption
Number of clinic visits for vaccine-related severe adverse event	1	1–2	Triangular	Assumption
**Utility inputs**				
Utility score of RSV uninfected	0.896	0.854–0.963	Beta	[29]
Utility score of self-managed care for RSV	0.82	0.73–0.94	Beta	[29]
Utility score of outpatient care for RSV	0.75	0.69–0.90	Beta	[29]
Utility score of hospitalization for RSV	0.576	0.560–0.592	Beta	[30]
QALY loss of severe adverse events	0.000677	0.000542–0.000812	Beta	[28]

RSV: respiratory syncytial virus; LRTD: lower respiratory tract illness; ARI: acute respiratory infection; QALY: quality-adjusted life-year; USD 1 = HKD 7.8.

### 2.5. Cost-Effectiveness Analysis and Sensitivity Analysis

All analyses were conducted in TreeAge Pro 2023 (TreeAge Software, Inc., Williamstown, MA, USA) and Microsoft Excel 365 (Microsoft Corporation, Redmond, WA, USA). A strategy was dominated (and, therefore, eliminated from the cost-effectiveness analysis) when it had a net loss of QALY at a higher cost (compared with another option). When a vaccination strategy demonstrated a net QALY gain with an incremental cost (versus the comparator), the incremental cost-effectiveness ratio (ICER) was calculated (ICER = Incremental cost/net QALY gain). A strategy was considered cost-effective if it resulted in (1) net QALY gain with a cost-saving, or (2) net QALY gain with an incremental cost and the ICER lower than the willingness-to-pay (WTP) threshold per QALY gained. The WTP threshold represents an estimate of the acceptable amount paid by the payer for the additional health benefit. As suggested by the World Health Organization, an ICER of less than 1× gross domestic product (GDP) per capita was considered highly cost-effective, and an ICER between 1–3× GDP per capita was considered cost-effective [35]. This study adopted the 1× GDP per capita as the WTP threshold, and the GDP per capita in Hong Kong was USD 49,594 in 2022 [36].

Sensitivity analyses were conducted to examine the robustness of the model results. One-way sensitivity analysis was performed on all parameters over the range for sensitivity analysis (Table 1) to identify influential factors. The parameters were varied over the upper and lower limits or the 95% CI. If not available, an assumed range formed by ±20% of the base-case value was made. The lower limit of 95% CI of VE of ABRYSVO^®^ in season 2 for LRTD and ARI were negative values, suggesting possible no protective benefit. A lower limit of zero was, therefore, applied for the range of sensitivity analysis of the VE of ABRYSVO^®^ in season 2. The RSV vaccines are yet to be marketed in Hong Kong and the pricing information is, therefore, unavailable. An extended one-way sensitivity analysis was, therefore, conducted on the vaccine price using USD 0 as the lower limit to examine the impacts of price variations. To evaluate the impact of uncertainty in all variables simultaneously, a Monte Carlo simulation was conducted in the probabilistic sensitivity analysis by randomly drawing each model input from the parameter-specific distribution (Table 1) and recalculating the direct cost and QALY loss for each intervention 10,000 times. The probabilities of each strategy over a range of WTP thresholds were presented in the acceptability curves.

## 3. Results

### 3.1. Base-Case Analysis

One set of expected outcomes (including event rates of RSV infection, RSV-associated hospitalization, and mortality) over two years in each study arm (Table 2) was generated by the decision tree model using the base-case value of the model inputs. The expected event rates in the ABRYSVO^®^ and AREXVY^®^ groups were lower than the expected event rates of the no-vaccination group. The expected event rates were the lowest in the ABRYSVO^®^ group.

The expected costs (at 4 levels of US vaccine price) and QALY loss per individual over two years are presented in Table 3. The expected QALY loss was lowest in the ABRYSVO^®^ group (0.001480 QALY loss), followed by the AREXVY^®^ group (0.001559 QALY loss) and the no-vaccination group (0.002127 QALY loss). When compared with no vaccination (as a common comparator), ABRYSVO^®^ and AREXVY^®^ gained 0.000647 QALY and 0.000568 QALY, respectively, and the ICERs (versus no vaccination) of ABRYSVO^®^ (26,209 USD/QALY gained) and AREXVY^®^ (47,485 USD/QALY gained) groups were accepted as cost-effective (less than the WTP threshold 49,594 USD/QALY gained) at the 25% US vaccine price level. At the 50%, 75%, and 100% US vaccine price levels, the ICERs of both vaccines exceeded the WTP threshold. 

Compared to the next costly option, the AREXVY^®^ group had a net QALY loss with higher cost versus the ABRYSVO^®^ group in all four price levels and was, therefore, dominated by the ABRYSVO^®^ group. At the 25% US vaccine price level, the ABRYSVO^®^ group (ICER 26,209 USD/QALY gained) was accepted as the cost-effective option. 

### 3.2. One-Way Sensitivity Analysis

**Figure 2:**
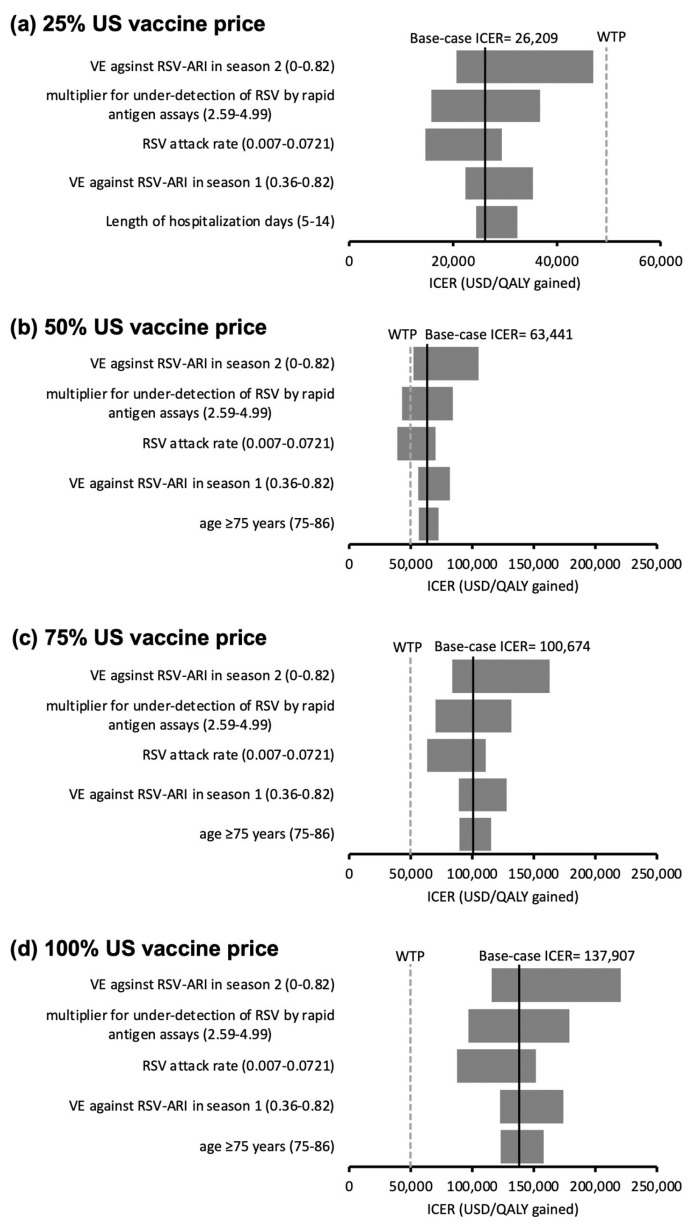
(**a**–**d**) Tornado diagrams of the variation of the ICER of the ABRYSVO^®^ group (versus no vaccination) against the top five influential parameters identified in one-way sensitivity analysis at (**a**) 25%, (**b**) 50%, (**c**) 75%, and (**d**) 100% US vaccine price levels. Ranges of variation in ICERs at 25% (**a**), 75% (**c**), and 100% (**d**) US vaccine price levels did not cross the WTP threshold (robust to the variation of model inputs). At 50% US vaccine price level (**b**), ICER < WTP threshold at variation of multiplier for under-detection of RSV by rapid antigen assays (base-case value 3.47) exceeded 4.38 or the RSV attack rate (base-case value 1.62%) exceeded 4.20%. US vaccine price: USD 200 for ABRYSVO^®^; RSV: respiratory syncytial virus; VE: vaccine efficacy; ARI: acute respiratory infection; LRTD: lower respiratory tract illness; ICER: incremental cost-effectiveness ratio; WTP: willingness-to-pay = 49,594 USD/QALY gained.

All the model inputs were assessed in the one-way sensitivity analyses. The top five influential factors on the ICER of ABRYSVO^®^ (versus no vaccination) are shown in the tornado diagrams (Figure 2a–d). The ranges of variation in ICERs of ABRYSVO^®^ at 25% (Figure 2a), 75% (Figure 2c), and 100% (Figure 2d) US vaccine price levels did not cross the WTP threshold, showing the results were robust to the variation of model inputs. At the 50% US vaccine price level (Figure 2b), the ICER of ABRYSVO^®^ became lower than the WTP threshold when the multiplier for the under-detection of RSV by rapid antigen assays (base-case value 3.47) exceeded 4.38 or the RSV attack rate (base-case value 1.62%) exceeded 4.20%. 

**Figure 3:**
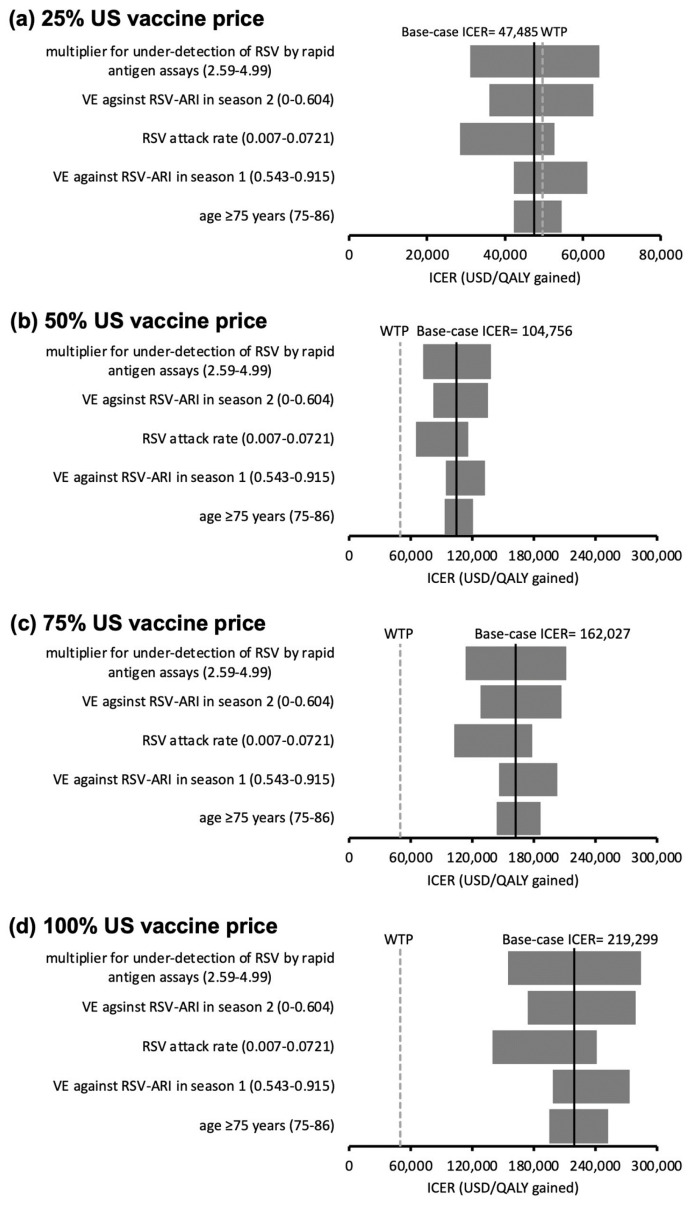
(**a**–**d**) Tornado diagrams of the variation of the ICER of the AREXVY^®^ group (versus no vaccination) against the top five influential parameters identified in one-way sensitivity analysis at (**a**) 25%, (**b**) 50%, (**c**) 75%, and (**d**) 100% US vaccine price levels. At 25% US vaccine price level (**a**), ICER > WTP threshold at variation of 14 influential factors (threshold values listed in Supplementary Materials Table S2). Ranges of variation in ICERs at the 50% (**b**), 75% (**c**), and 100% (**d**) US vaccine price levels did not cross the WTP threshold (robust to the variation of model inputs). US vaccine price: USD 270 for AREXVY^®^; RSV: respiratory syncytial virus; VE: vaccine efficacy; ARI: acute respiratory infection; LRTD: lower respiratory tract illness; ICER: incremental cost-effectiveness ratio; WTP: willingness-to-pay = 49,594 USD/QALY gained.

For the AREXVY^®^ group, 14 influential factors with threshold values were found at the 25% US vaccine price level. The ICER of AREXVY^®^ became higher than the WTP threshold when the values of the 14 influential factors crossed the threshold values listed in Supplementary Materials Table S2. The ICERs did not exceed the WTP threshold throughout one-way variation of all model inputs at the 50% (Figure 3b), 75% (Figure 3c), and 100% (Figure 3d) US vaccine price levels. The top five influential factors on the ICER of AREXVY^®^ (versus no vaccination) are shown in Figure 3a–d. 

The extended one-way sensitivity analysis of the vaccine price indicated that ABRYSVO^®^ would be cost-effective (versus no vaccination) when the cost per vaccination was USD 81 or less at a WTP threshold of 49,594 USD/QALY. AREXVY^®^ would be cost-effective (versus no vaccination) at the vaccination cost of USD 70 or less.

### 3.3. Probabilistic Sensitivity Analysis

**Figure 4:**
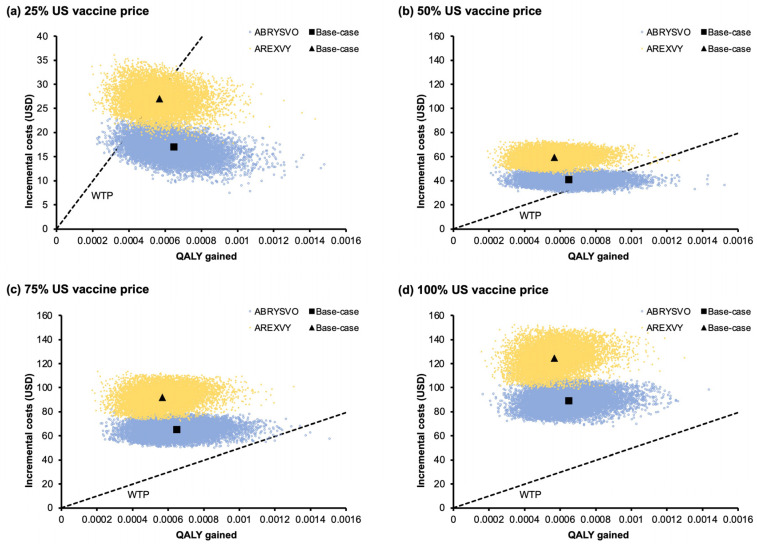
(**a**–**d**) Scatter plots of incremental cost and QALY gained by the vaccination strategies versus no vaccination in 10,000 Monte Carlo simulations at (**a**) 25%, (**b**) 50%, (**c**) 75%, and (**d**) 100% US vaccine price levels. ICERs of the ABRYSVO^®^ group (versus no vaccination) were below the WTP threshold in 97.66% (**a**), 15.77% (**b**), 0.07% (**c**), and 0% (**d**) of 10,000 simulations. ICERs of the AREXVY^®^ group (versus no vaccination) were below the WTP threshold in 53% (**a**), 0.05% (**b**), 0% (**c**), and 0% (**d**) of 10,000 simulations. US vaccine price: USD 270 for AREXVY^®^ and USD 200 for ABRYSVO^®^; QALY: quality-adjusted life year; WTP: willingness-to-pay; WTP = 49,594 USD/QALY gained.

The incremental costs and QALY gained by the ABRYSVO^®^ group and the AREXVY^®^ group versus the no-vaccination group in the 10,000 Monte Carlo simulations are shown in the scatter plots (Figure 4a–d). The ICERs of the ABRYSVO^®^ group (versus no vaccination) were below the WTP threshold in 97.66%, 15.77%, 0.07%, and 0% of the time at 25%, 50%, 75%, and 100% US vaccine price, respectively. The ICERs of the AREXVY^®^ group against no vaccination were below the WTP threshold in 53%, 0.05%, 0%, and 0% of simulations at 25%, 50%, 75%, and 100% US vaccine price levels, correspondingly. The mean (and 95% CI) additional costs and net QALY gained incurred by each vaccine in 10,000 simulations are listed in Table 4. Both the ABRYSVO^®^ and AREXVY^®^ groups exhibited statistically significant higher costs and lower QALY loss when compared to the no-vaccination group (*p* < 0.01).

**Figure 5:**
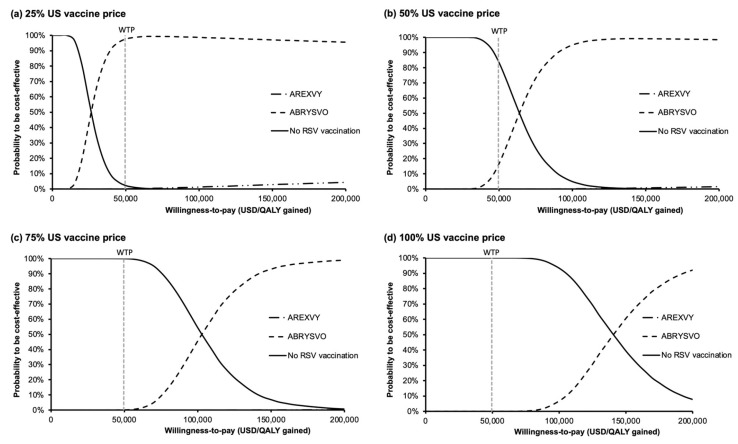
Variation in the probability of each vaccination strategy to be cost-effective against willingness-to-pay at (**a**) 25%, (**b**) 50%, (**c**) 75%, and (**d**) 100% US vaccine price levels. At the WTP threshold of 49,594 USD/QALY gained, probabilities of ABRYSVO^®^ to be the most cost-effective were 97.68% (**a**), 16.65% (**b**), 0.08% (**c**), and 0% (**d**). Probabilities of AREXVY^®^ to be the most cost-effective were 0.10% (**a**), 0% (**b**), 0% (**c**), and 0% (**d**). US vaccine price: USD 270 for AREXVY^®^ and USD 200 for ABRYSVO^®^; QALY: quality-adjusted life year; WTP: willingness-to-pay; WTP = 49,594 USD/QALY gained.

The acceptability curves (Figure 5a–d) were used to examine the probabilities to be cost-effective against a wide range of WTP (0–200,000 USD/QALY gained) for each group. At the WTP threshold of 49,594 USD/QALY gained, the probability of ABRYSVO^®^ to be the most cost-effective option was 97.68%, 16.65%, 0.08%, and 0% at 25%, 50%, 75%, and 100% US vaccine price levels, respectively. The probability of AREXVY^®^ to be the most cost-effective option was 0.10%, 0%, 0%, and 0% at 25%, 50%, 75%, and 100% US vaccine price levels, correspondingly. The probabilities of ABRYSVO^®^ to being the most cost-effective option were the highest of the three groups at WTP > 26,345, 63,847, 102,815, and 140,876 USD/QALY gained at 25%, 50%, 75%, and 100% US vaccine price levels, respectively.

## 4. Discussion

This is the first study to evaluate the potential cost-effectiveness of a single dose of two RSV vaccines to older adults aged 60 years and older in Hong Kong over a two-year period. AREXVY^®^ is an adjuvanted RSV prefusion F protein vaccine, and ABRYSVO^®^ is a bivalent RSV prefusion F vaccine. In the base-case analysis, the expected rates of RSV infection, RSV-associated hospitalization/mortality, and QALY loss in both AREXVY^®^ and ABRYSVO^®^ groups were lower than those of the no-vaccination group. The ICER (26,209 USD/QALY) of the ABRYSVO^®^ group (versus no vaccination) was found to be cost-effective (ICER < WTP 49,594 USD/QALY gained) when the vaccine price was set as 25% of the US vaccine price, and the results were robust to variations of model inputs. The acceptability curves of probabilistic sensitivity analysis further demonstrated that the ABRYSVO^®^ group showed the highest probability (97.68%) to be the most cost-effective strategy (at WTP 49,594 USD/QALY gained) at 25% US vaccine price. For the AREXVY^®^ group, the ICER (versus no vaccination) (47,485 USD/QALY gained) was marginally lower than the WTP threshold at 25% US vaccine price, and it was highly sensitive to the variation of model inputs in the one-way sensitivity analysis. The acceptability curves of probabilistic sensitivity analysis also indicated a low probability (0.10%) to be the most cost-effective strategy at 25% US vaccine price. 

In a prior cost-effectiveness analysis of a hypothetical RSV vaccine (with assumed VE 50%) for older adults in the US (RSV attack rate of 1.40–5.00%), the hypothetical vaccine was accepted as cost-effective at the WTP threshold of 50,000 USD/QALY gained if the vaccine price was less than USD 74–152 per vaccination [37]. A cost-effectiveness analysis of a hypothetical RSV with assumed VE 40% for older adults in the Netherlands (RSV attack rate of 3.32%) and the UK (RSV attack rate of 7.13%) reported that the vaccine was cost-effective if the vaccine price per individual was less than EUR 50.03 (at the WTP threshold of EUR 50,000 per QALY gained) and GBP 109.74 (at the WTP of threshold GBP 30,000/QALY gained), respectively [25]. Recently, the US CDC reported the economic evaluation of AREXVY^®^ and ABRYSVO^®^ in older adults over 2 years. The ICERs of the ABRYSVO^®^ (vaccine price of USD 200) and the AREXVY^®^ (vaccine price of USD 270) were 94,673 USD/QALY and 167,310 USD/QALY in the US older adults, respectively [27]. In the present study, one-way sensitivity analysis findings indicated that ABRYSVO^®^ and AREXVY^®^ were cost-effective (at WTP 49,594 USD/QALY) if the vaccine price per vaccination were less than USD 81 and USD 70 in Hong Kong older adults (RSV attack rate 1.62%; adjusted attack rate by multiplier: 3.55%). The findings of the prior studies were consistent with the present results that the cost-effectiveness of RSV vaccines was sensitivity to the vaccine price, RSV attack rate, and vaccine efficacy. 

As indicated in the one-way sensitivity analysis, higher RSV attack rates and higher multipliers for the under-detection of RSV by rapid antigen assays (for correction of reported RSV detected by rapid antigen assays) were influential factors with threshold values for the ICER of ABRYSVO^®^ to become acceptable as cost-effective at the 50% US vaccine price level. At higher RSV attack rates, the RSV infection cases preventable by the vaccine were increased and sequentially led to higher reduced cases of RSV-associated hospitalizations, deaths, and QALY loss (thus, higher net QALY gain). The reduced RSV cases consequently lowered the cost of RSV events, and, therefore, narrowed down the incremental cost between vaccinated populations versus those with no vaccination. The enhanced net gain of QALY with a reduced total cost, and, therefore, lower the ICER. 

RSV season typically occurs annually during the winter months, overlapping with influenza season. Co-administration of RSV and influenza vaccines, therefore, provides coverage on the period of shared seasonality of influenza and RSV [38,39]. It also allows healthcare providers to optimize and streamline vaccination strategies to increase vaccine coverage [40,41]. Phase three clinical trials of RSV and influenza vaccines co-administration, reported at the ACIP meeting, showed that it was well-tolerated with immune responses non-inferior to sequential administration [15,16]. The co-administration of RSV and influenza vaccines holds promise in reducing the clinical and economic burden of respiratory infections in older adults, and a cost-effectiveness analysis is warranted to inform the public healthcare provider.

The model-based study had several limitations. The search for model inputs was not conducted by a systematic review, and relevant data sources might, therefore, be missed. Overseas data were adopted for some model inputs when local data were not available. It might affect the generalization of model results to Hong Kong populations. The use of multipliers to adjust for the under-detected RSV events introduced uncertainty into the model outputs. VE data of RSV vaccines in year two post-vaccination (reported in the ACIP meeting 2023) were yet to be published in a peer-reviewed journal, contributing to the uncertainty in the VE values. The vaccine price levels in the model were based on the US price, due to the lack of Hong Kong-based RSV vaccine pricing information. The symptoms of RSV-associated ARI vary from mild localized symptoms (nasal congestion, cough, sneezing) to systemic symptoms (fever and myalgia). The utility of self-managed care (0.82) might overestimate the burden of QALY loss related to self-managed RSV in mild cases. We therefore conducted a sensitivity analysis on all model inputs using wide ranges of uncertainty to identify key influential parameters on the cost-effectiveness of RSV vaccines in Hong Kong. The paucity of Hong Kong epidemiology data on RSV burden in high-risk subgroups limited the model structure in that the hypothetical cohort was not differentiated into high-risk and non-high-risk groups at the entry, and the impacts of infection, hospitalization, and mortality rates within the high-risk group were not considered. The model timeframe was 2 years, limited by the short-term clinical data of VE. Long-term VE data is highly warranted for assessing the long-term cost-effectiveness of RSV vaccines. The present model is a static model and does not include the outcomes generated by the protection from herd immunity. Indirect costs, such as loss of productivity, were not included in the model. The present findings might, therefore, underestimate the benefits of RSV vaccination in older adults.

## 5. Conclusions

In conclusion, a single vaccination of ABRYSVO^®^ or AREXVY^®^ to adults aged 60 years and older appears to gain QALYs by reducing RSV-associated events over 2 years from the perspective of Hong Kong public healthcare providers. The cost-effectiveness of ABRYSVO^®^ or AREXVY^®^ is highly subject to vaccine price and RSV attack rate. 

## Figures and Tables

**Table 2:** Expected RSV infection, RSV-associated hospitalization, and mortality in the 2-year period post-vaccination (per 10,000 older adults).

Strategy	RSV Infection	RSV-Associated Hospitalization	RSV-Associated Mortality
No vaccination	707.4	28.290	2.183
AREXVY^®^	516.3	20.647	1.593
ABRYSVO^®^	491.9	19.671	1.518

RSV: respiratory syncytial virus.

**Table 3:** Expected direct costs and QALY loss per individual of each vaccination strategy in base-case analysis.

Strategy	Direct Costs (USD)	QALY Loss	ICER vs. Next Less Costly Option	ICER vs. No Vaccination
25% US vaccine price *				
No vaccination	24	0.002127	-	-
ABRYSVO^®^	41	0.001480	**26,209**	**26,209**
AREXVY^®^	51	0.001559	dominated	**47,485**
50% US vaccine price *				
No vaccination	24	0.002127	-	-
ABRYSVO^®^	89	0.001480	63,441	63,441
AREXVY^®^	116	0.001559	dominated	104,756
75% US vaccine price *				
No vaccination	24	0.002127	-	-
ABRYSVO^®^	89	0.001480	100,674	100,674
AREXVY^®^	116	0.001559	dominated	162,027
100% US vaccine price *				
No vaccination	26	0.002127	-	-
ABRYSVO^®^	113	0.001480	137,907	137,907
AREXVY^®^	149	0.001559	dominated	219,299

* US vaccine price: USD 270 for AREXVY^®^ and USD 200 for ABRYSVO^®^; RSV: respiratory syncytial virus; QALY: quality-adjusted life-year; ICER: incremental cost per QALY gained; ICER vs. next less costly option = (Total cost strategy − Total cost next less costly strategy)/(QALY loss next less costly strategy − QALY loss strategy); ICER vs. no vaccination = (Total cost strategy − Total cost no vaccination)/(QALY loss no vaccination − QALY loss strategy); Bold: ICER < Willingness-to-pay threshold (49,594 USD/QALY gained).

**Table 4:** Incremental costs and QALY gained by vaccination strategies versus no vaccination in a probabilistic sensitivity analysis.

Strategy	Net QALY Gained, Mean (95% CI) ^a^
ABRYSVO^®^	0.000645 (0.000642–0.000648)
AREXVY^®^	0.000565 (0.000562–0.000567)
	**Incremental Costs (USD), Mean (95% CI) ^a^**
25% US vaccine price	
ABRYSVO^®^	16.66 (16.62–16.70)
AREXVY^®^	26.97 (26.92–27.03)
50% US vaccine price	
ABRYSVO^®^	40.65 (40.58–40.73)
AREXVY^®^	59.38 (59.28–59.48)
75% US vaccine price	
ABRYSVO^®^	64.84 (64.73–64.95)
AREXVY^®^	92.01 (91.86–92.16)
100% US vaccine price	
ABRYSVO^®^	89.13 (88.98–89.28)
AREXVY^®^	124.82 (124.62–125.02)

US vaccine price: USD 270 for AREXVY^®^ and USD 200 for ABRYSVO^®^; QALY: quality-adjusted life-year; ^a^ All *p*-values < 0.01.

## Data Availability

All data used in this study were obtained from published literature and public data. All data generated or analyzed during this study are included in this published article as Supplementary Materials files.

## Supplementary Materials

The following supporting information can be downloaded at: https://www.mdpi.com/article/10.3390/vaccines11101605/s1. Table S1: Literature search strategy of MEDELINE; Figure S1: Flow diagram of literature search and selection process for clinical inputs; Table S2: Threshold values of influential parameters on the ICER of AREXVY^®^ (versus no vaccination) in one-way sensitivity analysis at 25% US vaccine price level.
