# Supplementary material for: Comparative Cost-Effectiveness Analysis of Respiratory Syncytial Virus Vaccines for Older Adults in Hong Kong
Source: Vaccines (Basel). 2023 Oct 17;11(10):1605. doi: 10.3390/vaccines11101605 (PMC10610694; doi:10.3390/vaccines11101605)
Supplement: Supplementary file 1 [file vaccines-11-01605-s001.zip › vaccines-2619810-supplementary.pdf]

Supplementary materials

Title: Comparative cost-effectiveness analysis of respiratory syncytial virus vaccines for older adults in Hong Kong

Table S1. Search strategy of MEDELINE

Figure S1. Flow diagram of literature search and selection process for clinical inputs

Table S2. Threshold values of influential parameters on the ICER of AREXVY® (versus no vaccination) in one-way sensitivity analysis at 25% US vaccine price level

Table S1. Search strategy of MEDELINE

#	Query
1	(aged or homes for the aged or elderly or geriatr* or older patient* or old patient* or older person* or old person* or older adult* or old adult* or adult*).mp.
2	(vital statistics or morbidity or mortality or incidence or prevalence or hospital* or general practitioner* or disease burden).mp.
3	(respiratory syncytial virus vaccine* or respiratory syncytial virus, human or human respiratory syncytial virus or rsv or bronchiolitis).mp.
4	1 and 2
5	3 and 4
6	(China or Chinese or Hong Kong or Taiwan or Macao).mp.
7	5 and 6
8	limit 7 to (English language and yr="2000 - 2023")

* is a symbol that broadens a search term by finding words that start with the same letters

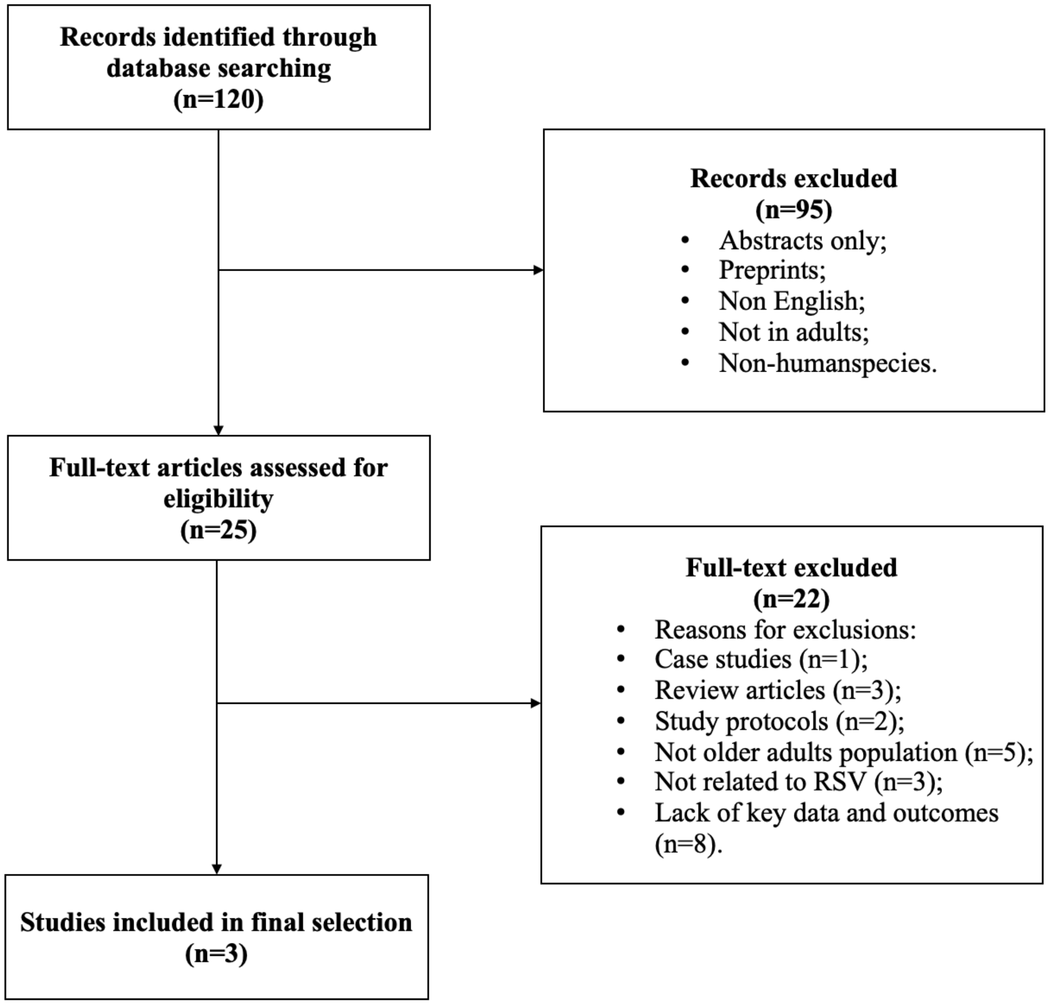

Figure S1. Flow diagram of literature search and selection process for clinical inputs

Table S2. Threshold values of influential parameters on the ICER of AREXVY® (versus no vaccination) in one-way sensitivity analysis at 25% US vaccine price level

Parameters	Base-case value	Threshold value*
Multiplier for under-detection of RSV by rapid antigen assays	3.47	<3.35
Vaccine efficacy against RSV-ARI in season 2	27.8%	<23.08%
RSV attack rate	1.62%	<1.23%
Vaccine efficacy against RSV-ARI in season 1	79%	<74.43%
Age ≥75 years	80	>81.88
RSV mortality rate (per 10,000 population) in ≥75 years	0.8263	<0.7610
Length of hospitalization days	12	<9.64
Utility score of self-managed care for RSV	0.82	>0.88
Vaccine efficacy against RSV-LRTD in season 2	52.9%	<29.22%
Utility score of RSV uninfected	0.896	<0.866
Utility score of outpatient care for RSV	0.75	>0.81
Proportion of RSV-ARI among RSV infections	84.2%	>93.39%
RSV mortality rate (per 10,000 population) in 65-74 years	0.1423	<0.1155
Vaccine efficacy against RSV-LRTD in season 1	87.5%	<64.61%

RSV: respiratory syncytial virus; ARI: acute respiratory infection; LRTD: lower respiratory tract illness.

*The ICER (base-case value= 47,485 USD/QALY gained) exceeded the WTP threshold (49,594 USD/QALY gained) when the parameters crossed the threshold values.
